# Influence of Spin Coating Parameters on Gas Transport Properties of Thin-Film Composite Membranes

**DOI:** 10.3390/ma14175093

**Published:** 2021-09-06

**Authors:** Stepan Sokolov, Alexey Balynin, Danila Bakhtin, Ilya Borisov

**Affiliations:** A.V. Topchiev Institute of Petrochemical Synthesis RAS, Russian Academy of Sciences, 117912 Moscow, Russia; sokolovste@ips.ac.ru (S.S.); ab@ips.ac.ru (A.B.); db2@ips.ac.ru (D.B.)

**Keywords:** spin coating, PTMSP, thin-film composite membranes, polymer penetration

## Abstract

The influence of casting centrifugation process parameters, such as a rotation speed (ω), the amount of the film-forming solution (V), and its concentration (C) on transport properties of composite membranes were investigated. A number of composite membranes based on poly (1-trimethylsilylpropyne) (PTMSP) and micro- (MFFK-1) and ultrafiltration (UFFK) membranes were obtained using the spin-coating method. For the first time, an unexpected dependence of permeance and ideal selectivity on rotation speed had been discovered: the thickness of the selective layer decreases from 3.0 to 1.0 μm for MFFK-1 and from 1.7 to 1.1 μm for UFFK with an increase of spin coater rotation speed from 500 to 3000 rpm. However, the gas permeance of composite membranes in the range of 500–2000 rpm was reduced due to an increase of a penetration depth of PTMSP into a support layer porous structure (estimated by the EDX method). The permeance of the PTMSP/UFFK membranes was higher than PTMSP/MFFK-1 membranes due to a thinner selective layer and a lower penetration depth of polymer solution into the pores of the support. The highest CO_2_/N_2_ selectivity values were achieved as 5.65 ± 0.9 at CO_2_ permeance 5600 ± 1000 GPU for PTMSP/UFFK membranes (C_PTMSP_ = 0.35%, V_solution_ = 1 mL, ω = 1000 rpm), and 6.1 ± 0.5 at CO_2_ permeance 4090 ± 500 GPU for PTMSP/MFFK-1 membranes (C_PTMSP_ = 0.35%, V_solution_ = 1 mL, ω = 2000 rpm).

## 1. Introduction

Due to its high gas permeability, a high free volume glassy polymer poly (1-trimethylsilylpropyne) (PTMSP) is often used to form a gutter layer in ultrathin film composite (TFC) membranes for gas separation [1,2,3,4]. A gutter layer considerably improves TFC membrane performance preventing the penetration of the selective polymer into the porous support [5]. One of the most common methods of film composite membranes production is the dip-coating method: porous support is immersed in a film-forming solution of a polymer for a certain time, and then removed from it. In dependence on the adhesive interaction and the viscosity of the film-forming solution, a layer of the solution remains on the surface of the support, which, after evaporation of the solvent, forms a thin layer of the selective polymer. Using the dip-coating method flat [6,7], hollow fiber [8,9], and tubular [10,11] membranes can be obtained.

In previous work, we showed that the deposition of a thin top-layer of PTMSP on the porous PAN-support from its 0.5 wt % solution in chloroform by using the kiss-coating technique was accompanied by the intrusion of polymeric solution into the pores [12]. It is important to emphasize that the penetration depth of PTMSP was comparable to the thickness of the top-layer. The influence of the porosity of the support on the gas transport properties of the composite membrane has been studied in a number of works [13,14,15]. In the study [16] composite membranes Pebax-1657/PEI and Pebax-1657/PES with a selective layer with thicknesses of 1.4 and 1.7 μm, respectively, reduced their permeances from expected 46 and 38 GPU to 17 and 27 GPU due to the penetration of the polymer into the pores of the supports.

A less common, but very perspective approach of flat composite membranes preparation is the spin-coating method. In a spin coating process, the porous support spins around the vertical axis, and the film-forming solution is applied to a support perpendicular to its surface during rotation. By varying the spin coating parameters (the amount of the film-forming solution, its concentration, the rotation speed, and the spin time), it is possible to control the thickness of the resulting layer of the selective polymer.

Le Roux and Paul were among the first to use spin-coating to create composite gas separation membranes [17]. In their study, poly-4-methyl-1-pentene (PMP) was deposited onto the surface of the supports by spin coating method from its solutions in cyclohexane. Two types of supports were used in this work: a porous asymmetric polysulfone (PSF) membrane and a composite membrane with a thin layer of polydimethylsiloxane (PDMS) with a thickness of 1.7 μm deposited on the same porous PSF support. It was shown that for composite membranes PSF/PMP without an intermediate layer of PDMS, it was necessary to apply several layers of PMP to obtain a defect-free selective layer.

In recent years, spin-coating has been successfully used to obtain composite membranes with thin separation layers based on hybrid polymer materials with the addition of three- [18,19,20] and two-dimensional nanoparticles [21,22,23,24]. Thus, in [21], composite membranes with thin selective layers of poly [1-trimethylsilyl-1-propyne] (PTMSP) with additions (1 wt %) of graphene (G) and graphene oxide (GO) were obtained. A porous polypropylene film with slit pores (0.117 µm × 0.042 µm) was used as a support. The selective layers were deposited using PTMSP solutions in chloroform. Multilayer graphene particles had a transverse size of 5 μm and a thickness of 2–8 nm, while single-layer graphene oxide nanoplates had a transverse size of 2 μm and a thickness of 1.1 nm. Seven samples of PTMSP/G membranes with a selective layer thickness of 1.6–7.0 µm and 6 PTMSP/GO samples with a selective layer thickness of 1.6–6.2 µm were obtained and studied. It is important that, despite the large transverse size of the graphene fillers, all membranes with a selective layer thickness of 1.6 to 7 μm turned out to be defect-free. This confirms that the graphene plates (G and OG) are oriented parallel to the horizontal surface of the film.

However, the authors of [21] did not analyze the issue of polymer penetration into the porous structure of the support layer. The goal of this study was to determine the dependence of gas transport properties of PTMSP composite membranes and its morphology (thickness of the selective layer and penetration depth) on such spin coating parameters as rotation speed (ω), amount (V), and concentration (C) of film-forming solution. Two types of porous supports were used as support layer: microfiltration (MFFK) and ultrafiltration (UFFK) membranes made of fluoroplastic F-42L. The porous structure of the supports and composite membranes was analyzed by scanning electron microscopy (SEM). The thickness of the selective PTMSP layer was determined from the SEM data, and the depth of PTMSP penetration into the pores of the support layer was determined by energy-dispersive X-ray elemental spectroscopy (EDX). The gas transport properties of the membranes were investigated by the volumetric method for two gases-carbon dioxide (CO_2_) and nitrogen (N_2_).

## 2. Materials and Methods

### 2.1. Chemicals

Commercially available membranes were used as porous supports for composite membranes: microfiltration MFFK-1 and ultrafiltration UFFK membrane manufactured by Vladipor (Vladimir, Russia). MFFK-1 and UFFK represent a porous layer of fluoroplastic F-42L, applied to a nonwoven fabric made of polyethylene terephthalate. Synthesis of PTMSP (catalytic system TaCl_5_/Al (i-C_4_H_9_)), used for selective layer formation, was carried out at the Laboratory of Synthesis of Selectively Permeable Polymers (TIPS RAS) and described in [25].

### 2.2. Membrane Fabrication

PTMSP was deposited on porous supports from polymer solutions in chloroform with mass concentrations C = 0.17, 0.25, 0.35, and 0.50 wt %. The viscosity of the solutions was measured using a Brookfield DV2T-RV viscometer (Ametek Brookfield, Middleboro, MA, USA) at a temperature of 25 °C. For the application of PTMSP solutions onto porous supports, the spinNXG-P1A (Apex Instruments, Kolkata, India) was used. The system was equipped with a foreline pump for support fixing. Porous supports MFFK-1 and UFFK were attached to a flat glass plate using adhesive tape. The polymer solution (0.5, 1, 1.5, or 2 mL) was fed from a pipette to the center of the rotating support. In this study, the rotation speed varied from 500 to 3000 rpm.

### 2.3. Gas Permeance Measurement

Gas transport characteristics of the obtained composite membranes were measured within the day after the deposition of the selective layer. The single gas permeance of the samples Q was measured by the volumetric method (N_2_ first, CO_2_ second). The excess pressure in the experiments Δp varied from 1 to 2 bar, while atmospheric pressure was maintained on the permeate side. The working surface of the membrane S was 12.6 cm^2^. The gas flow through the membrane was measured with a flow meter. Membrane permeance expressed in GPU units was estimated by the linear extrapolation of experimental data to zero trans-membrane pressure.

(1) $$Q=\frac{P}{l}=\frac{V}{\mathrm{tS}\Delta p}$$

where V–the volume of the gas passing through the membrane per unit of time t.

The selective properties of the membrane were estimated by the value of the ideal selectivity α_CO_2_/N_2__ as the ratio of permeabilities of individual gases

(2) $$\alpha_{{\mathrm{CO}}_{2}/N_{2}}=\frac{P_{{\mathrm{CO}}_{2}}}{P_{N_{2}}}$$

To obtain the dependence of the gas permeance on the volume of film-forming solution at a constant rotation speed (750 rpm), 0.5, 1, 1.5, and 2.0 mL of PTMSP solution were applied on the supports. The spinning time was 120 *s*, which was sufficient for complete evaporation of the solvent. To determine the dependence of gas permeance on the rotation speed, the following values were investigated: 500, 750, 1000, 1500, 2000, 2500, and 3000 rpm. For each mode, at least three samples were prepared and measured to control the convergence of the results.

### 2.4. Scanning Electron Microscopy (SEM) and Energy-Dispersive X-ray Elemental Spectroscopy (EDX)

The morphology of the samples of supports and composite membranes (surface and transverse cleavages) was studied using a scanning electron microscope Thermo Fisher Phenom XL G2 Desktop SEM (Thermo Fisher Scientific Inc., Waltham, MA, USA), equipped with a module for energy-dispersive elemental spectroscopy (EDX). Using a magnetron sputter Cressington 108 auto Sputter Coater (Cressington Scientific Instruments UK, Watford, UK) a thin layer of gold 5–10 nm was applied to the surface of the samples. The value of the accelerating voltage during the measurement was 15 keV. The determination of the average thickness of the selective layer, as well as the surface porosity of the supports from the obtained micrographs, was carried out using the Gwyddion software (ver. 2.53). For each composite membrane, three images of a transverse cleavage were taken from different regions of the sample. Five measurements of the thickness of the selective PTMSP layer (l_top_) were taken for each of the micrographs. The penetration depth of PTMSP into the porous structure of the support (l_sup_) was estimated by EDX according to the method, based on the signal strength of the silicon (Si) atom in the near-surface layer and proposed earlier [12].

## 3. Results

**Figure 1:**
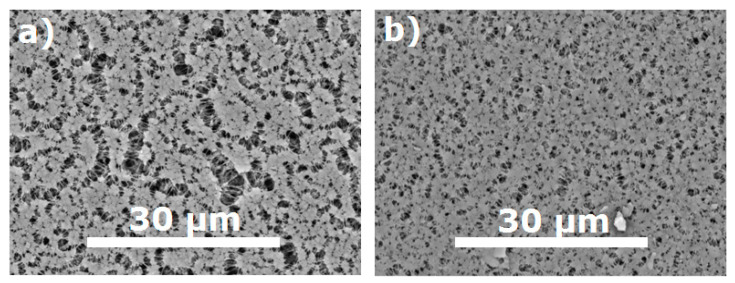
SEM images of MFFK-1 (**a**) and UFFK (**b**) surfaces.

**Figure 2:**
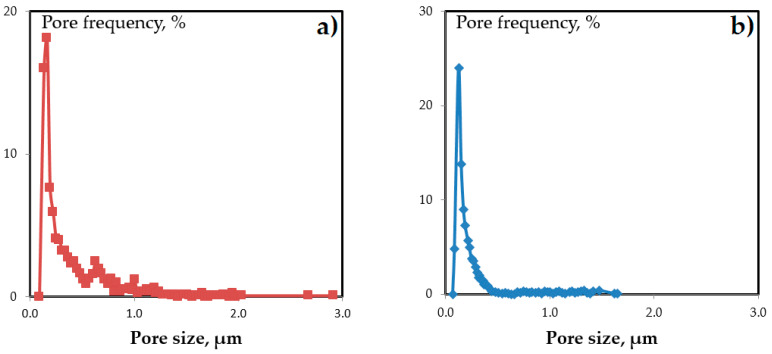
MFFK-1 (**a**) and UFFK (**b**) pore size distribution.

Since the porous support can play a major role in the formation of thin top-layer, ultra- (UFFK) and microfiltration (MFFK-1) membranes made of the same polymeric material fluoroplastic F-42L were used in this work. The SEM images of the top-surface of UFFK and MFFK-1 membranes presented in Figure 1 were analyzed by using the Gwyddion software (ver. 2.53) that enabled to obtain the size distributions of surface pores (Figure 2a,b). Table 1 lists the corresponded data on surface porosity, average and modal (most probable) pore sizes for both porous support.

It can be seen from Table 1 that the average pore size of MFFK-1 was about 290 nm in contrast to 190 nm for UFFK, and the difference in the surface porosity was about twice, 66% and 35%, respectively. It is important to emphasize that the pore size distribution of the MFFK-1 (see Figure 2b) revealed the presence of the larger pores up to 3 μm. Moreover, the estimated pore fraction in the range from 0.5 to 0.7 μm was 12.5% and 0.9% for MFFK-1 and UFFK, respectively. Bearing in mind higher surface porosity of the MFFK-1, penetration of PTMSP solution into support layer can be expected under the same applying conditions in contrast to UFFK support. As reported in [15], CO_2_ permeance was equal to 137,800 and 67,500 GPU, and the ideal CO_2_ /N_2_ selectivity was 0.88 and 0.91 for MFFK-1 and UFFK, respectively. Such high gas permeance values allowed to neglect the resistance of the support layer despite the fact that even fibrous porous media with big pores could affect the gas transport as reported recently [26].

In this study, the PTMSP concentration in the casting solution was varied from 0.17 up to 0.5 wt %. The viscosities of PTMSP film-forming solutions in chloroform are presented in Table 2. It can be seen that with an increase in the concentration of the polymer in the solution from 0.17% to 0.50%, its viscosity increased by a factor of nearly 30, which makes it possible to control the viscosity characteristics of the film-forming solution during the preparation of composite membranes.

### 3.1. Volume of the Film-Forming Solution

**Figure 3:**
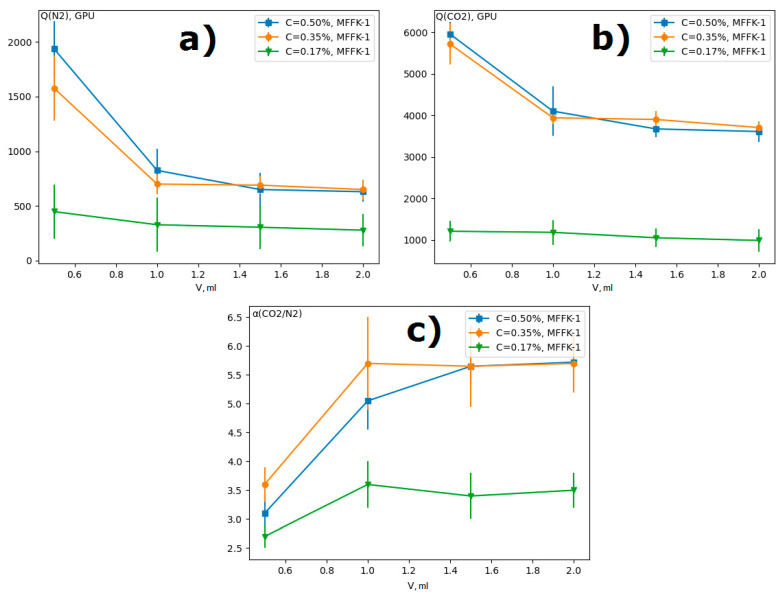
Gas transport properties of composite MFFK-1/PTMSP membranes obtained by applying PTMSP solutions of various concentrations, depending on the volume of the solution (ω = 750 rpm): (**a**) N_2_ permeance, (**b**) CO_2_permeance, (**c**) ideal CO_2_/N_2_ selectivity.

The dependence of gas transport properties of composite membranes on the volume of the applied PTMSP solution for various concentrations is shown in Figure 3.

**Figure 4:**
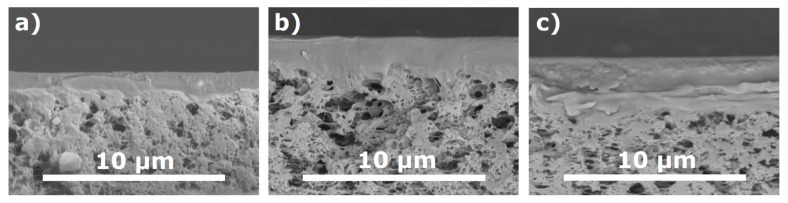
SEM image of a transverse cleavage of PTMSP/MFFK-1 membranes obtained by applying different volumes of PTMSP solution (0.50 wt %) at a rotation speed of 750 rpm: (**a**) 1.0 mL, (**b**) 1.5 mL and (**c**) 2.0 mL.

The permeance of N_2_ and CO_2_ decreased with the increase of the applied solution amount for three studied PTMSP solutions. Figure 4 shows micrographs of a transverse cleavage of PTMSP/MFFK-1 composite membranes obtained by applying different volumes of PTMSP solution with a concentration of 0.50 wt % at a rotation speed of 750 rpm. The thickness of the selective layer l_top_ was 1.4 ± 0.4, 2.7 ± 0.5, and 3.7 ± 0.7 μm for composite membranes obtained with 1.0, 1.5, and 2.0 mL of PTMSP solution, respectively. The decrease in the permeance of composite membranes with the increase of applied PTMSP solution amount (Figure 3a,b) can be explained by the increment of selective layer thickness (Figure 4). It can be seen that the resulted thickness of the top-layer was not linearly corresponded with the volume of polymeric solution used for spin-coating. For instance, the thickness of the top-layer obtained by using 1 mL of solution was 1.4 μm, whereas the increase of the amount of the same solution up to 2 mL resulted in the formation of top-layer having a thickness of 3.7 μm. Such behavior can be explained by the partial intrusion of the polymeric solution into the pores of the support at the beginning of its deposition; the ratio of the polymer penetrated into the pores and remained on the surface would be greater for a smaller quantity of polymeric solution.

It was previously shown that dense membranes with a thickness 30–40 µm from this PTMSP sample demonstrate an ideal CO_2_/N_2_ selectivity of 5.6 [27]. As can be seen from Figure 3c, the application of 0.5 mL of film-forming solution did not provide the formation of a selective layer with sufficient selectivity. For further research, the volume of the PTMSP solution of 1 mL was chosen, at which the high selectivity and gas permeance had been achieved.

### 3.2. Concentration of the Film-Forming Solution

Table 3 shows gas transport properties of composite PTMSP/MFFK-1 membranes obtained by applying PTMSP solutions of various concentrations. The permeance of composite membranes formed using PTMSP solutions with a concentration of 0.17 and 0.25 wt % was significantly lower than the corresponding values for membranes obtained from solutions of 0.35 and 0.50 wt % for all rotation speeds studied. In this regard, for further studies, only 0.35 and 0.50 wt % PTMSP solutions were used. The high permeance of composite membranes obtained from solutions with high polymer concentration (0.35 and 0.50 wt %) may be due to their high viscosity (Table 2). This kind of dependence of permeance of composite membranes on the viscosity of the film-forming solution was observed earlier [28]. In the study [28] PDMS/ceramic composite membranes were prepared using 7.5 wt % PDMS solutions in n-heptane with various prepolymerization times, which directly affects the viscosity of the solution by the dip-coating method. The authors of this work noted, that with an increase in viscosity of the film-forming solution, the gas permeance of composite membranes increases due to the inhibition of polymer penetration into the porous structure of the ceramic membrane. However, not for all composite membranes this kind of dependence was observed. Thus, in [29], it was shown that for composite PDMS/PVDF membranes, an increase in viscosity, on the one hand, prevented the penetration of the film-forming solution into the pores, but, on the other hand, it helped to form a dense selective layer on the surface of the support. As a result, the pervaporation performance of PDMS/PVDF composite membranes in 1.0 wt % n-butanol/water mixtures had an extreme dependence on the viscosity of PDMS solution in n-heptane with a maximum in the selectivity value at a viscosity of 45 cP.

According to the literature data and results obtained within the work, the more viscous polymer solution could be less prone to penetration into the porous structure of the supports. The viscosity of the PTMSP film-forming solution applied to the porous MFFK-1 support by a spin-coating method should be at least 80 cP to prevent severe penetration and to form a high-permeable selective layer.

### 3.3. Rotation Speed

The thickness of the selective layer of composite membranes obtained by deposition of PTMSP solution in chloroform (C = 0.35%) on porous supports of MFFK-1 and UFFK at different rotation speeds is presented in Table 4. An increase in the rotation speed led to a decline in the thickness of the selective layer l_top_ of the PTMSP/MFFK-1 and PTMSP/UFFK composite membranes. The inversely proportional dependence of the layer thickness on the rotation speed was described back in 1958 by Emsley, Bonner, and Peck [30], considering the problem of the motion of a viscous non-volatile fluid on an infinite rotating disk. However, for the thicknesses of polymer films obtained from film-forming solutions by spin coating, similar dependence was also observed [31,32].

It should be noted that the l_top_ value for the PTMSP/MFFK-1 membrane was approximately two times higher than for the PTMSP/UFFK membrane at a rotation speed of 500 rpm. This was apparently due to the greater roughness of the surface of the MFFK-1 support. The difference in l_top_ values reduced for MFFK-1 and UFFK-based membranes and became equal to about 1 μm when approaching rotation speeds of the order of 3000 rpm.

**Figure 5:**
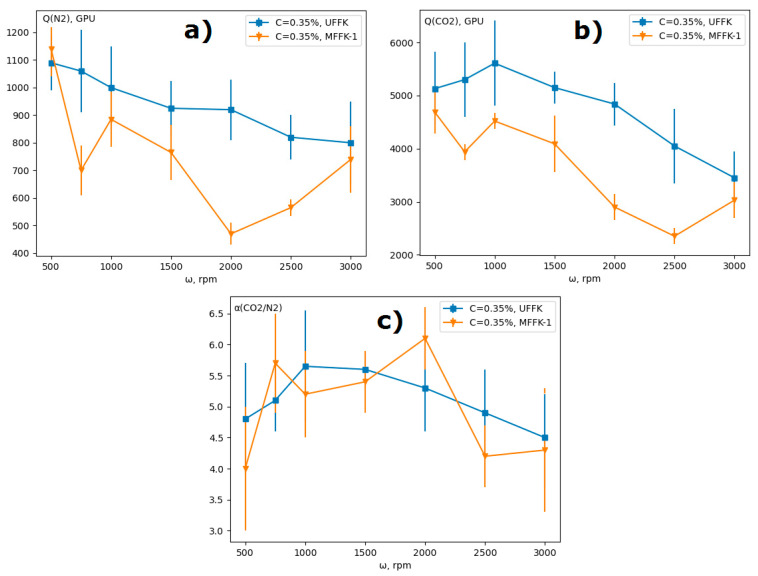
Gas transport properties of composite membranes obtained by applying PTMSP solution (C = 0.35 wt %) on MFFK-1 and UFFK supports at different spin coater rotation speeds: (**a**) N_2_ permeance; (**b**) CO_2_permeance; (**c**) ideal CO_2_/N_2_ selectivity.

**Figure 6:**
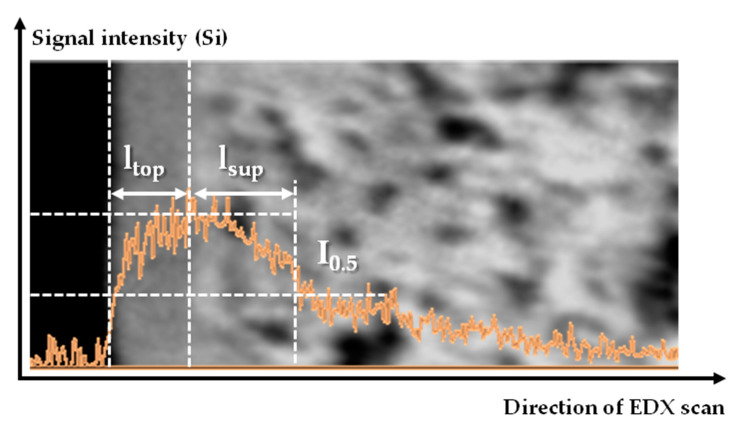
Estimation of PTMSP penetration depth into the pores of the support (l_sup_).

Based on these results, one could expect an increase in the gas permeance of composite membranes with an increase in the rotation speed during formation. However, a decline was observed in the gas permeance of N_2_ and CO_2_ with the rotation speed increase in the range of 500–2000 rpm (Figure 5). This can be explained by partial penetration of the film-forming solution into the pores of the support, estimated by EDX method (Figure 6). As follows from Table 5, the PTMSP penetration depth in the support l_sup_ was comparable (two times less) with the value of the selective layer l_top_ (Table 4). For example, the l_sup_ and l_top_ values were 1.6 and 3.0 μm for the PTMSP/MFFK-1 membrane obtained at 500 rpm. Moreover, the value of the PTMSP penetration depth l_sup_ increased with the increment of the rotation speed from 500 to 3000 rpm for MFFK-1 and UFFK. It is important to note that the polymer penetration depth into the UFFK pore structure was approximately 1.5 times less than that for the MFFK-1. Thus, the total thickness of the PTMSP layer in the PTMSP/UFFK composite membranes was less than that for the PTMSP/MFFK-1 membranes. As a result, the gas permeance of the PTMSP/UFFK membranes was higher (Figure 5a,b).

To summarize, two opposite effects acted on the dependence of gas permeance and selectivity on rotation speed: the thickness of the selective layer reduces, according to the EBP equation, however the penetration depth of PTMSP increases. At a rotation speed more than 2500 rpm, the selective layer became too thin and does not provide formation sufficient selectivity. As a result, the CO_2_/N_2_ selectivity had a maximum value in 1000 and 2000 rpm for UFFK and MFFK-1, respectively, and CO_2_ permeance for MFFK-1 had a minimum at 2500 rpm.

Under the same formation conditions, the permeability of PTMSP membranes based on less porous UFFK (surface porosity is 35%) was higher than for MFFK-based (66%) membranes, and the penetration of PTMSP into the porous structure was less pronounced. Thus, TFC with better gas transport characteristics could be obtained with a smoother and less permeable membrane (e.g., UFFK) as support for the spin-coating formation method.

Unfortunately, the spin-coating method did not reduce the penetration depth of PTMSP into the porous support (with an average pore size of 190 and 290 nm) and the gas permeance of the samples obtained by this method (4000–6000 GPU) was lower than that of the samples obtained by the kiss-coating method (13,000–15,000 GPU) with the same supports [16], but ideal values of CO_2_/N_2_ selectivity were higher and closer to the selectivity of dense PTMSP membranes. However, these results can be utilized towards fabrication of multilayer TFC membranes for CO_2_ capture with the gutter layer made of a polymer of intrinsic microporosity like PTMSP.

## 4. Conclusions

In this work, defect-free composite membranes with PTMSP selective layer on micro- (MFFK-1) and ultrafiltration (UFFK) membranes (“Vladipor”, Russia) were obtained by the spin coating method. The dependence of N_2_ and CO_2_ transport properties of composite membranes and its morphology on parameters of spin coating process was found.

With an increase in the concentration of the film-forming solution, the gas permeance of the thin-film composite membranes grew due to the increase PTMSP solution viscosity, which prevents the penetration of the polymer into the porous structure of the support. The highest gas permeance and selectivity were demonstrated by samples obtained from PTMSP solutions in chloroform with concentrations C = 0.35 wt % and 0.50 wt %. An increase of the solution volume consumed for one spin-coating application led to the decrease of membranes gas permeance, while the values of the ideal selectivity reach a plateau at 1 mL of film-forming solution or more. With an increase in the rotation speed, the unexpected dependence of the gas permeance of N_2_ and CO_2_ was observed. In the range of rotation speed from 500 to 2000 rpm the gas permeance decreases, despite the reduction of selective layer thickness. This effect can be explained by the PTMSP penetration depth into the porous structure of the support, which increased with rotation speed. The highest CO_2_/N2 selectivity was achieved with following parameters of the selective layer application: C = 0.35%, V = 1 mL, ω = 1000/2000 rpm for UFFK and MFFK-1, respectively; and was equal to α_CO2/N2_ = (5.65 ± 0.9) at CO_2_ permeance (5600 ± 1000) GPU for PTMSP/UFFK membranes and α_CO2/N2_ = (6.1 ± 0.5) with CO_2_ permeance (4090 ± 500) GPU for MFFK-1.

## Figures and Tables

**Table 1:** Surface porosity of MFFK-1 and UFFK.

Sample	Average Pore Size, nm	Modal Pore Size, nm	Surface Porosity, %
MFFK-1	290	160	66
UFFK	190	130	35

**Table 2:** Dynamic viscosity of the polymer solutions.

PTMSP Concentration, wt %	Dynamic Viscosity, cP
0.17	11 ± 3
0.25	34 ± 3
0.35	80 ± 3
0.50	300 ± 25

**Table 3:** Gas transport properties of composite PTMSP/MFFK-1 membrane obtained by applying PTMSP solutions of various concentration (ω =1000 and 1500 rpm).

Concentration, wt %	Rotation Speed, rpm	N_2_ Permeance, GPU	CO_2_ Permeance, GPU	Ideal Selectivity CO_2_/N_2_
0.17	500	220 ± 20	970 ± 100	4.4 ± 1.0
	1000	130 ± 20	520 ± 100	4.0 ± 0.5
	3000	230 ± 30	870 ± 100	3.8 ± 0.6
0.25	500	290 ± 50	1560 ± 150	5.4 ± 0.9
	1000	125 ± 20	575 ± 150	4.0 ± 0.5
	3000	250 ± 50	1020 ± 200	4.1 ± 0.5
0.35	500	1140 ± 100	4690 ± 400	4.1 ± 1.0
	1000	875 ± 100	4520 ± 100	5.2 ± 0.7
	3000	740 ± 120	3130 ± 340	4.2 ± 1.0
0.50	500	910 ± 150	5040 ± 500	5.5 ± 0.5
	1000	890 ± 150	4550 ± 200	5.2 ± 1.0
	3000	970 ± 100	4400 ± 150	4.5 ± 0.5

**Table 4:** Thickness of the outer selective layer (l_top_) of composite membrane obtained by applying PTMSP solutions in chloroform (0.35 wt %) on porous supports MFFK-1 and UFFK at different rotation speeds.

Rotation Speed, rpm	l_top_, µm	
	PTMSP/MFFK-1	PTMSP/MFFK
500	3.0 ± 0.5	1.7 ± 0.2
750	2.5 ± 0.2	1.6 ± 0.2
1000	2.2 ± 0.2	1.3 ± 0.2
1500	2.1 ± 0.8	1.3 ± 0.3
2000	1.6 ± 0.3	1.3 ± 0.1
2500	1.3 ± 0.4	1.2 ± 0.2
3000	1.0 ± 0.2	1.1 ± 0.2

**Table 5:** Estimated by EDX PTMSP penetration depth into the pores of the support (l_sup_) for composite membranes obtained by applying PTMSP solution in chloroform (C = 0.35 wt %) at different rotation speed.

Rotation Speed, rpm	l_sup_, µm	
	PTMSP/MFFK-1	PTMSP/UFFK
500	1.6	0.9
1000	1.4	1.6
3000	1.9	1.1

## Data Availability

Data will be made available upon reasonable request.
